# Modulation of memory reconsolidation by adjacent novel tasks: timing defines the nature of change

**DOI:** 10.1038/s42003-023-05666-5

**Published:** 2023-12-19

**Authors:** Matías Nicolás Schroeder, Camila L. Fullio, Fabricio Ballarini, Diego Moncada

**Affiliations:** 1 Laboratorio de Neurofisiología de la Memoria, Instituto de Biología Celular y Neurociencia, Facultad de Medicina (UBA/CONICET) - Instituto Tecnológico de Buenos Aires (ITBA), Ciudad Autónoma de Buenos Aires, Argentina; 2 Laboratorio de Neurofisiología de la Memoria, Instituto de Biología Celular y Neurociencia “Prof. E. De Robertis” (IBCN), Facultad de Medicina, UBA-CONICET, Ciudad Autónoma de Buenos Aires, Argentina; 3 Laboratorio de neurociencia translacional, Instituto de Biología Celular y Neurociencia “Prof. E. De Robertis” (IBCN), Facultad de Medicina, UBA-CONICET, Ciudad Autónoma de Buenos Aires, Argentina; 4 Instituto Tecnológico de Buenos Aires, Av. Madero 399, Ciudad Autónoma de Buenos Aires, Argentina; 5 Centro Integrativo de Biología y Química Aplicada, Universidad Bernardo O’Higgins, Santiago, Chile

**Keywords:** Learning and memory, Hippocampus, Long-term memory

## Abstract

Reconsolidation turns memories into a responsive state that allows their modulation until they stabilize again. This phenomenon attracted remarkable attention due to its potential impact on therapeutics and education. Recent evidence revealed that different memories undergo reconsolidation via a behavioral tagging process. Thus, their re-stabilization involves setting “reconsolidation-tags” and synthesizing plasticity-related proteins for their capture at the tagged sites. Here, we studied the possibility of affecting these fundamental mechanisms to modulate reconsolidation. Our findings, in laboratory rats, indicate that exploring a novel environment 60 min before or after memory reactivation improves spatial object recognition memory by promoting protein synthesis. Conversely, experiencing novelty immediately after reactivation impairs the reconsolidation by affecting the tags. Similar effects, but with a different optimal time window for improvement, occur in inhibitory avoidance memory. These results highlight the possibility of modulating existing memories using non-invasive interventions that selectively affect the fundamental mechanisms of behavioral tagging during their reconsolidation.

## Abstract (web summary)

A study on rats shows how to modulate memories by associating their recall with non-invasive behavioral experiences: timing defines the nature of change acting on the key mechanisms of the behavioral tagging process underlying memory reconsolidation.

## Introduction

Memory, the ability to codify, store and retrieve information, allows numberless animals to shape their behavior in response to previous experience, therefore playing a critical role in the multiple facets of life. The stabilization of fragile and transient memory traces into stable ones is a crucial phase in long-term memory (LTM) formation. This process is known as consolidation^1^. However, adapting a learned behavior to new circumstances, like those arising from environmental changes, results as crucial as the original learning. Memory reconsolidation is a mechanism proposed to account for this updating^2–7^. This process, triggered upon certain reminder events, destabilizes a LTM that must go through a new consolidation-like period for its restabilization^8,9^. During this time, the memory becomes labile and susceptible to interferences and may thus allow changes to update the original mnemonic trace. Like memory consolidation, its reconsolidation also depends on protein synthesis to be successful^9^. In the same direction, recent evidence shows that both phases of memory rely on a ‘behavioral tagging’ process^10^.

The behavioral tagging hypothesis was proposed as a behavioral analog of the synaptic tagging and capture hypothesis^11,12^, formulated by Frey and Morris to explain the synaptic specificity in models of functional plasticity, such as long-term potentiation and depression^13,14^. The behavioral tagging hypothesis proposes a process that explains the stimulus input specificity during the formation of lasting memories^15^. It postulates two parallel and complementary mechanisms acting during LTM formation: the setting of transient learning-tags upon learning, establishing a potential neuronal substrate for storing recently acquired information, and the synthesis of plasticity-related proteins (PRPs) that allow memory consolidation upon capture at the tagged sites^12,15,16^.

The behavioral tagging hypothesis provides a framework to predict under which circumstances discrete events interact with each other, resulting in the induction, impairment, or modulation of lasting memories. Thus, experiences capable of providing PRPs to available learning tags, set by a weak training session usually unable to induce protein synthesis, can promote the consolidation of a lasting memory that otherwise would not exist^16–18^. In fact, the behavioral tagging process’s existence was shown in such a way, associating a weak inhibitory avoidance (IA) training with the exploration of a novel open field^11^. Moreover, events providing further PRPs to the available tags may induce stronger memories, while experiences competing for those proteins or disrupting putative leaning tags may impair lasting memories^19,20^.

A behavioral tagging process underlying memory reconsolidation implies that, during this phase, the fate of memory would be subjected to its rules^10^. Thus, events capable of changing the availability of proteins or the functionality of the learning-tags during the reconsolidation could alter the reactivated memories^21^. Therefore, different behavioral experiences occurring in the temporal proximity of a memory-reactivating event might be used as non-invasive strategies to improve or attenuate already established memories, depending on their effect over the proteins’ availability or the integrity of the ‘reconsolidation-tags’.

This work directly addresses these possibilities in two different learning-tasks in rodents: the IA and the spatial version of the object recognition task (SOR). It reveals that the delayed association of a novel experience to a memory-reactivating event can improve that memory by providing extra PRPs to those required for its reconsolidation. Moreover, impairing the memory labilization induced by the reactivating event also blocked this improving effect. In contrast, when the novel experience occurred immediately after the reactivating event, it impaired the reconsolidation tags and disrupted the original memory. In summary, we show how a behavioral event can modify the strength and integrity of previously consolidated memories by affecting the behavioral tagging process underlying their reconsolidation.

## Materials and Methods

### Animals

We used male Wistar rats (weight: 250–300 g, 2.5-month-old aprox.) from the Faculty of Medicine of the Buenos Aires University. Rats were housed in groups of three per cage, with water and food ad libitum, at a constant temperature of 23 °C and under a 12 h light/dark cycle (lights on: 7 a.m.). Behavioral procedures were conducted during the light phase.

The experimental protocols were approved by CICUAL (Institutional Committee for the Care and Use of Laboratory Animals) of the School of Medicine of Buenos Aires University: Resolution 294/2019. We have complied with all relevant ethical regulations for animal use.

### Surgery and drug infusion

Cannula implantation, drug infusion and histological examination of cannula placements were performed as described previously^11^. Briefly, guide cannuli were stereotaxicaly placed 1 mm above the pyramidal cell layer of the CA1 region of the dorsal hippocampus (DH) of deeply anesthetized rats, using the coordinates of the atlas of Paxinos and Watson as guide^22^. From bregma: Posterior −3.9 mm, Lateral ±3.0 mm, Ventral 3.0 mm. Rats were allowed to rest and recover for at least one week before any procedural manipulation. To infuse the drugs, a 30-gauge infusion needle with its tip protruding 1 mm beyond that of the guide was used. Only data from animals with correct cannula implants (>95 % of the rats) were included in the analyses.

### Drugs

All drugs were purchased from Sigma. Emetine (EME) (50 µg) and (2 *R*)-amino-5-phosphonovaleric acid (AP-V) (5 µg) were infused in volume of 0.8 µl (saline) per side to specifically inhibit protein synthesis and antagonize N-methyl-D-aspartate (NMDA) receptors. These doses proved to be effective previously^10,23,24^.

### Behavioral apparatus and procedures

To avoid unnecessary emotional stress, all rats were handled daily for 3 min during 3 days before any behavioral procedure. Then animals were randomly assigned to each experimental group/condition.

The open field (OF) apparatuses were squared (50 cm side) or circular (50 cm diameter) arenas with different colors and materials in their floors and walls (40 cm height). A novel environment exploration consisted of a 5 min exposure to either a squared or circular OF^11^.

For the IA we used a step-down IA system manufactured by Med Associates-inc. For the reconsolidation protocol, we subjected rats to two habituation sessions, a training session, a reactivation session, and a test session each of them spaced by 24 h. All sessions started by placing the rats in the platform placed on the left side of the box. During the habituation session, the rats explored freely the avoidance box for 5 min. In the training session, immediately after stepping down from the platform with their four paws, they received an electric foot shock (0.5 mA during 3 s). On day four, in the reactivation session, animals were placed on the platform and taken from it after 40 s. On day five, we performed a full test session by placing the animals on the platform and measuring the latency to step down. An increase in the step-down latency from training to test is indicative of memory. A greater latency is indicative of a better memory expression^10^.

The Spatial Object Recognition (SOR) apparatus was a 60 cm wide x 40 cm length x 50 cm high white acrylic box with a transparent front and different visual clues. For the first two days, we habituated the animals to the context by letting them explore the arena without objects for 30 min each day. On day three in the training phase, we placed two identical objects into the context and let the animals explore freely for 8 min. The next day, in the reactivation session, we switched one of the objects to a new position and allowed the rats to explore for 2 min. Finally, during the test session (24 h later), we moved the same object again to another new position and left the animals to explore one more time for 2 min. Since the training, we recorded the exploration time for each object in each session. Then, we calculated a discrimination index: (Time in novel - Time in old position)/(Time in novel + Time in old position). Positive values of this index reflect the presence of memory. Values around zero or equivalent to training indicate its absence^10^.

In order to reduce the use of animals, as a general rule rats were used twice, once for experiments involving the IA task and once for experiments involving the SOR task. Animals were allowed to rest for 1 week before starting the second experimental round^10,25^. IA and SOR behavior and performance were equivalent regardless they had performed the task in the first or second round of experiments.

The experimenter in charge of performing the behavioral measurements remained blinded to the experimental condition until the experiment finished.

### Statistics and reproducibility

Data are expressed as mean SEM and were analyzed using GraphPad Prism software. Samples were analyzed via one-way analysis of variance (ANOVA) or Welch’s ANOVA test for heteroscedastic samples. Multiple comparisons were corrected using the Sidak’s or the Tamhane T2 method, respectively. See figure legends for details. Differences were considered significant for α > 0.05. The discrimination indexes and step-down latencies were equivalent in all training groups of all the experiments. Thus, for the sake of simplicity, only one representative training group is shown in the figures. Sample size for each experiment was estimated by previous experience with different experiments.

### Reporting summary

Further information on research design is available in the Nature Portfolio Reporting Summary linked to this article.

## Results

**Fig. 1:**
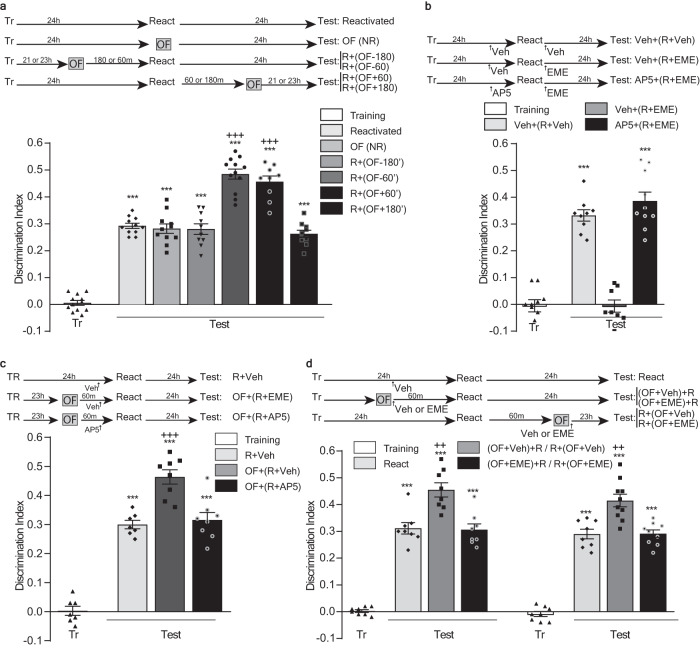
Peri-reactivation exposure to a novel OF improves SOR-LTM during its reconsolidation by a protein synthesis-dependent process. Top: experimental design. Figures show the discrimination index between the object moved to the novel position and the non-moved object, expressed as mean ± SEM, during training (Tr) and test session. R: animals submitted to a reactivation session. NR: animals not submitted to a reactivation session. **a**: *** *p* < 0.001 vs Tr and +++ *p* < 0.001 vs Reactivated, OF (NR), R + (OF-180) and R + (OF + 180), multiple comparisons after Welch’s ANOVA (*n* = 9–12); W_(11,44.26)_ = 156.2. **b**: ***p < 0.001 vs Tr and Veh + (R + EME), multiple comparisons after one-way ANOVA (*n* = 8–10); F_(5, 48)_ = 57.97. **c**: ***p < 0.001 vs Tr and +++ *p* < 0.001 vs R+Veh and OF + (R + AP5), multiple comparisons after one-way ANOVA (*n* = 7–8); F_(5, 40)_ = 123.2. **d**: ***p < 0.001 vs Tr and ++ *p* < 0.01 vs (OF + EME) + R or R + (OF + EME), multiple comparisons after Welch’s ANOVA (*n* = 8; W_(5, 21.43)_ = 151.6 for the left part and *n* = 8–10; W_(5, 19.25)_ = 104.7 for right the part).

First, we studied the possibility of up-modulating a reactivated memory through the association of another behavioral experience in the time frame of interaction with the behavioral tagging process underlying memory reconsolidation. To do this, we trained rats in the SOR task as described previously^10^, and 24 h later, distinct groups of animals were subjected to explore, or not, a novel OF at different times before or after submitting them to a SOR reactivation session. Finally, we evaluated the SOR memory after another 24 h. As shown in Fig. 1a, the exploration of the novel arena 60 min before or after the reactivation session resulted in improved LTM expression compared to those animals that did not explore it. The memory levels of the latter group were equivalent to those of animals that explored novel OF but 180 min before or after the reactivation session. It is worth noting that the improving effect relies on the novel nature of the arena since exploring a familiar OF, 60 min before the reactivation session, did not improve memory expression at the next day (Supplementary Fig. 1a. Supplementary Note 1).

Our previous work demonstrated that the OF exploration has no effect over the post-reactivation short-term memory^10^, indicating that the improvement effect shown above is specific over the post-reactivation LTM, established upon the reconsolidation process. However, to further assess this issue, we analyzed if such improvement depended on the memory labilization that characterizes the reconsolidation phase. We started studying the possibility of impairing SOR memory labilization by affecting NMDA receptors’ function during the reactivation session. To do this, we evaluated the effect of infusing the NMDA antagonist AP-V and/or the protein synthesis inhibitor EME in the DH. We observed that infusing EME immediately after the reactivation session impaired SOR-memory reconsolidation and induced long-term amnesia 24 h later. In contrast, the infusion of AP-V 15 min before the reactivation session did not affect memory expression the next day. However, infusing AP-V prevented the amnestic effect of applying EME (Fig. 1b), indicating the requirement of the NMDA function to labilize the memory trace and start the reconsolidation process. Then we evaluated if this labilization was a prerequisite to induce the memory improvement triggered by the exploration to the novel arena. To do that, we trained animals on the SOR task. During the next day, we let the rats explore a novel OF, or not, and after 60 min we submitted them to a SOR reactivation session performed under the previous infusion of AP-V or vehicle (Veh). When evaluating LTM expression 24 h later, we observed that the animals infused with Veh and that explored the novel OF performed better than those who did not explore it. More importantly, this improvement was absent in the group of animals submitted to the novel arena but infused with AP-V before the reactivation session (Fig. 1c).

A behavioral tagging process underlying memory reconsolidation implies that after its reactivation, the interaction of the learning-tags with the available proteins will determine the resultant memory trace. Since setting the learning-tags does not require protein synthesis, but it relies on the experience that triggers the mnemonic process, we reason that the improving effect of the exploration to the novel arena should result from its capacity to synthesize proteins, beyond those synthesized by the reactivation session, which would then be available for capturing by de SOR-reconsolidation tag. To evaluate this possibility, we trained rats in the SOR task, and the next day, 60 min before or after a SOR reactivation session we allowed them to explore a novel OF, or not, and infused them with EME or Veh, in the DH immediately after the OF exploration. Finally, we evaluated the LTM expression 24 h later. Confirming the previous results, the animals infused with Veh that explored the novel arena, regardless of they did it before or after the reactivation session, improved SOR-LTM expression compared to those that did not explore it. More importantly, in both time points, the administration of EME impaired this improvement effect, and the memory expression levels remained comparable to those of animals only subjected to the reactivation session (Fig. 1d).

**Fig. 2:**
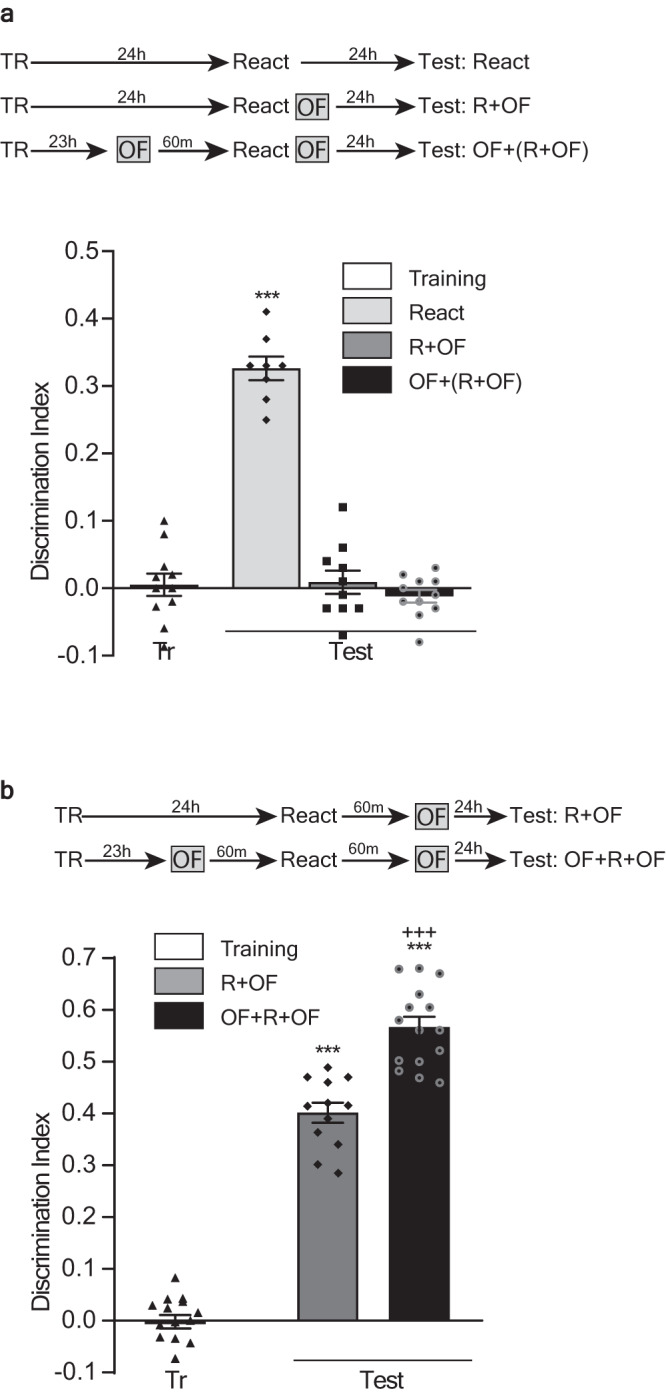
Novelty impairs or improves SOR-LTM during reconsolidation depending on its proximity to the reactivation session. Top: experimental design. Figures show the discrimination index between the object moved to the novel position and the non-moved object, expressed as mean ± SEM, during training (Tr) and test session. R: animals submitted to a reactivation session. NR: animals not submitted to a reactivation session. **a**: ***p < 0.001 vs all groups, multiple comparisons after one-way ANOVA (*n* = 8–11); F_(5, 52)_ = 62.04. **b**: ***p < 0.001 vs Tr and +++*p* < 0.001 vs R + OF, multiple comparisons after one-way ANOVA (*n* = 12–15); F_(3, 50)_ = 309.1.

Beyond the effect on the PRPs synthesis process, depending on the moment they occur, the association of multiple experiences may interact by affecting the stability of the learning-tags established by each experience. Previous evidence suggests that OF exploration close to a training session may impair the SOR ‘learning-tags’^11,18^. Thus, to evaluate if this occurs during the reconsolidation of this memory, we trained rats in the SOR task, and thereupon 24 h we subjected them, or not, to explore a novel OF right after the reactivation session. We also used a third group of animals that explored two different and novel OF, one 60 min before the reactivation session and the other immediately after it. As shown in Fig. 2a, the exposure to the novel arena immediately after the reactivation session, far from improving SOR-LTM, impaired the reconsolidation process leading to long-term amnesia 24 h later. In addition, the previous exposure to another novel OF, 60 min before de reactivation session, did not prevent this amnestic effect. As shown above, the novelty experienced at this moment improved the reconsolidated memory through a process dependent on protein synthesis (Fig. 1a, d). Therefore, the result suggests that the novel experience occurring immediately after the reactivation session impaired the SOR reconsolidation-tags, preventing the ulterior capture of proteins of any source, and leading to long-term amnesia instead of memory improvement. Confirming this view, animals exposed to the second OF 60 min after the reactivation session, rather than immediately after it, not only exhibited SOR-LTM the next day, they outperformed those animals that only explored a single novel OF 60 min before the reactivation (Fig. 2b). In fact, we also observed an incremental effect on memory improvement when exposing animals to the novel OF for 2 or 5 min, two exploration times that detect spatial novelty^26,27^. Each exposure, performed 60 min before a reactivation session, improved memory expression the next day. Yet, the 5 min group outperformed the 2 min group (Supplementary Fig. 2a. Supplementary Note 2).

**Fig. 3:**
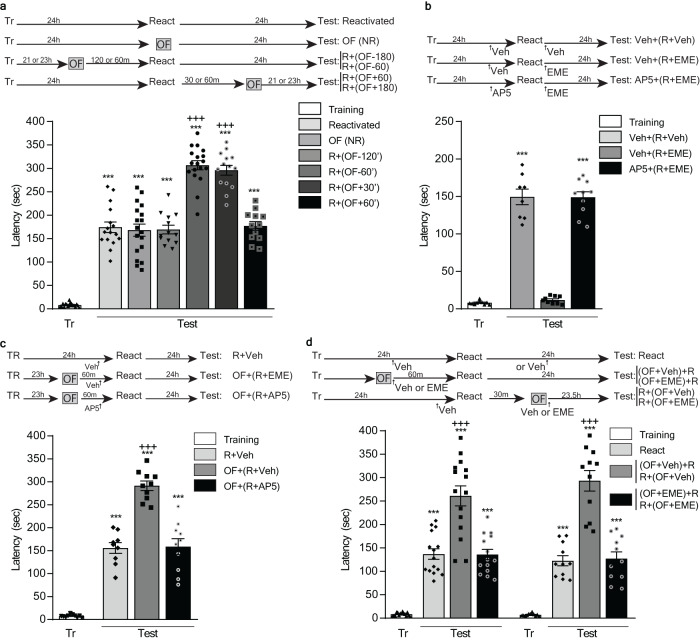
Peri-reactivation exposure to a novel OF improves IA-LTM during its reconsolidation by a protein synthesis-dependent process. Top: experimental design. Figures show latency to step-down from the platform during training (Tr) and test session, expressed as mean ± SEM. R: animals submitted to a reactivation session. NR: animals not submitted to a reactivation session. **a**: ***p < 0.001 vs Tr and +++ *p* < 0.001 vs Reactivated, OF (NR), R + (OF-120) and R + (OF + 60), multiple comparisons after Welch’s ANOVA (*n* = 12–18); W_(11, 64.13)_ = 222.9. **b**: ***p < 0.001 vs Tr and Veh + (R + EME), multiple comparisons after Welch’s ANOVA (*n* = 8–10); W_(5, 21.7)_ = 97.57. **c**: ***p < 0.001 vs Tr and +++*p* < 0.001 vs R+Veh and OF + (R + AP5), multiple comparisons after Welch’s ANOVA (*n* = 9–10); W_(5, 23.23)_ = 177.7. **d**: ***p < 0.001 vs Tr and +++*p* < 0.001 vs (OF + EME) + R or R + (OF + EME), multiple comparisons after Welch’s ANOVA (*n* = 14–15; W_(5, 29.91)_ = 64.46 for the left part and *n* = 11; W_(5, 36.75)_ = 75.66 for the right part).

**Fig. 4:**
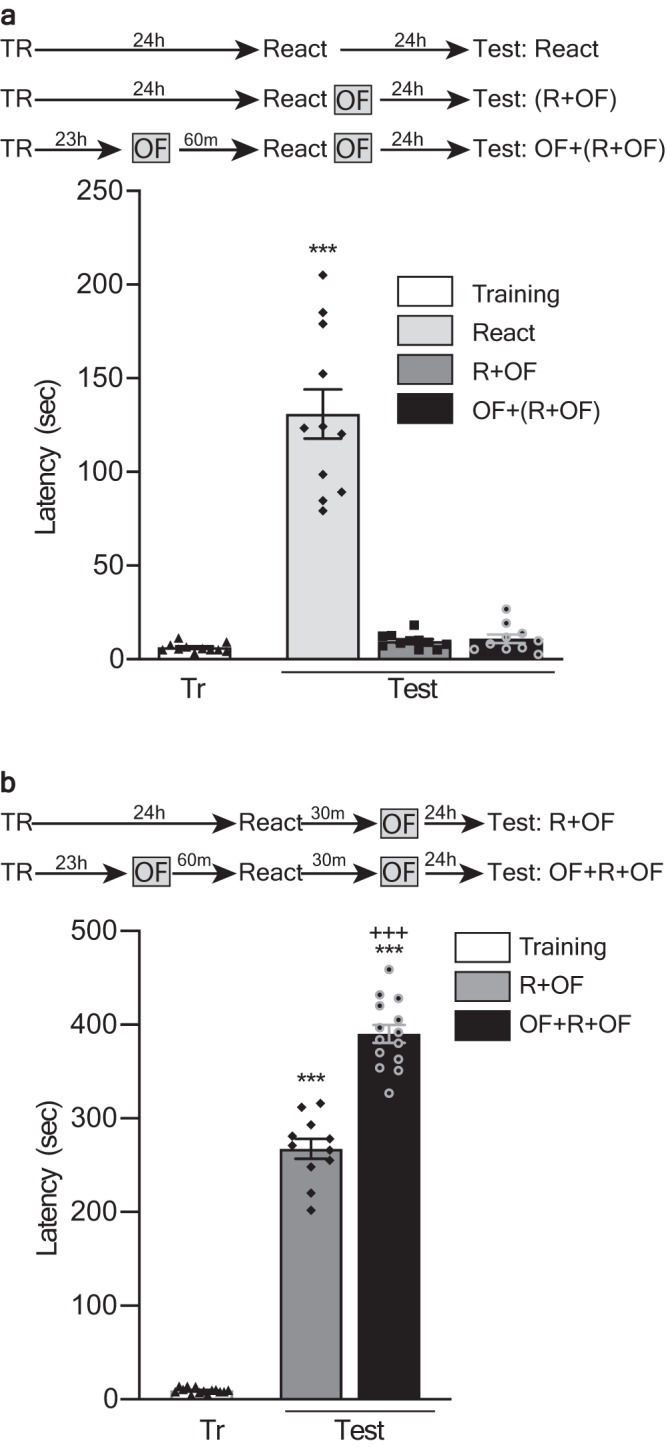
Novelty impairs or improves IA-LTM during reconsolidation depending on its proximity to the reactivation session. Top: experimental design. Figures show latency to step-down from the platform during training (Tr) and test session, expressed as mean ± SEM. R: animals submitted to a reactivation session. NR: animals not submitted to a reactivation session. **a**: ***p < 0.001 vs all groups, multiple comparisons after Welch’s ANOVA (*n* = 10–11); W_(5, 26.72)_ = 18.22. **b**: ***p < 0.001 vs Tr and +++*p* < 0.001 vs R + F, multiple comparisons after Welch’s ANOVA (*n* = 11–14); W_(3, 22.1)_ = 663.3.

Since evidence points to the behavioral tagging as a general mechanism underlying the formation of lasting memories, we next evaluated the flexibility of acting over this process to improve or impair distinct memories during their reconsolidation. To do so, we repeated the previous series of experiments in a different learning task, the IA. Unlike the SOR, which is a spatial learning task, the IA is an operant-like conditioning, which as well as the SOR and the OF exploration is processed, among other structures, in the hippocampus^28–31^. As shown in Fig. 3a, the animals that explored a novel OF 60 min before or 30 min after an IA-reactivation session improved their LTM-expression 24 h later, compared to animals that did not explore it. Besides, the level of performance of the last ones was equivalent to those of animals that explored the novel arena beyond this time window. Also, the improving effect was observed if animal explored a novel OF 60 min before the reactivation session, but not if they explored a familiar one (Supplementary Fig. 1b. Supplementary Note 1). Once more, preventing memory labilization via AP-V infusion (Fig. 3b) impaired the improving effect of the exploration to the novel arena (Fig. 3c). In line with the previous results and the Behavioral Tagging hypothesis, impairing the protein synthesis following the OF exploration, either before or after the IA reactivation session, also compromised its improving effect on memory (Fig. 3d). Again, exploring the OF next to the reactivation session induced retroactive amnesia, which the previous exploration to a different and novel OF could not prevent (Fig. 4a). Also, exploring the second arena 30 min after the reactivation, improved the reconsolidated memory beyond the levels induced by the exploration of a single novel OF explored 60 min before the reactivation session (Fig. 4b). Finally, exploring the novel OF for 5 min improved the reconsolidated memory to a higher level of performance than doing it for 2 min (Supplementary Fig. 2b. Supplementary Note 2). These results highlight for the second time that novel experiences associated with a memory reactivation event can provide PRPs to improve the reconsolidation of memories, but also to impair the reconsolidation-tags and disrupt the LTM.

## Discussion

In this work, we studied the effect of novel behavioral experiences associated with a memory retrieving event on the behavioral tagging process that underlays memory reconsolidation. We showed that exploring a novel OF within a critical time window around a SOR- or an IA- reactivation session resulted in an improved LTM. This effect depended on the synthesis of new proteins triggered upon the OF exploration, and it was fostered by the exploration of a second novel OF within the permissive time window. Moreover, we found that the same novel experience occurring immediately after the reactivation session induced long-term retrograde amnesia by affecting a protein synthesis independent process, like the reconsolidation-tag.

Different works studied the possibility of modulating memory during its reconsolidation phase. Most of them focused on understanding the effects of reconsolidation on established memories and showed, in different cases, that the sole reactivation could serve as a memory enhancer. This type of effect was found in rodents and humans^32–37^. Instead of focusing on the effect of reconsolidation on memory, we studied how experiences occurring close to a reactivating event act on the reconsolidation process to affect previously stored memories. Here, we showed that exploring a novel, but not familiar, arena close to a memory reactivation session improved its LTM expression 24 h later. The effect befell when the novelty was experienced 1 h, but not 3 h, before or after the reactivation of SOR memory; or between 1 h before to 30 min after the reactivation of IA memory. These time windows match those observed in a previous study showing that exploring a novel arena could provide the proteins required to reconsolidate a memory reactivated in the presence of a protein synthesis inhibitior^10^. That work showed that the reconsolidation of SOR and IA memory occurs through a behavioral tagging process, suggesting that the improving effect observed in this work also relies on this mechanism.

Wang^21^ suggested a similar idea after showing that exploring a novel box close to the reactivation of an event memory, or a fear conditioning memory, improved their persistence. Here, we did not study the duration of memories but their expression at a fixed time. Yet, we did focus on evaluating the mechanism altered by the events associated with the reactivation of memory to modulate its reconsolidation. We provide different pieces of evidence to support our postulate that positive and negative effects on the reactivated memory are the result of the interaction between the processes triggered by the associated event and the behavioral tagging process underlying SOR and IA memories reconsolidation.

The fundamental nature of a behavioral tagging process stands on the ability of learning-tags, set upon a particular experience, to capture PRPs to stabilize the mnemonic trace^12,16^. Since proteins may result limiting in different conditions, the availability of additional proteins to capture may well determine the consolidation of memories or plastic changes at a functional plasticity level, as well as their strength^19,20^. Here, the reconsolidation-tags set upon memory-reactivation might have also captured proteins synthesized by the novel event to reinforce memory. In fact, inhibiting protein synthesis after exploring the novel arena impaired its improving effect. On the contrary, we showed that exploring two different and novel OFs, within the critical time window, improved SOR and IA memories expression further than exploring a single arena. Again, this suggests that the availability of additional proteins enhanced their reconsolidation.

On 2016, Nomoto and colleagues showed that the promoting effect of novelty acting on the behavioral tagging process during an object recognition (OR) memory consolidation relayed not only in the synthesis of new proteins but also in the level of overlap between the neuronal populations activated by the SOR learning and the exploration to the novel arena. This overlapping was higher than the one observed when the animals explored a familiar environment, which did not promote OR-memory consolidation through behavioral tagging^38^; a lack of effect also shown in different learning tasks^11,18^. Also, Guzowski and colleagues showed that exploring different novel arenas activates shared and non-shared neuronal populations^39^. In addition, we show here that exploring a familiar OF fails to improve memory during its reconsolidation, thus highlighting again the importance of the novel nature of the arena. Together, these results suggest that the additional reinforcement of reconsolidation induced when the animals explore a second novel environment may be a consequence of inducing protein synthesis in a neuronal substrate associated with the reconsolidation processing but not targeted by the first novelty. Also, the possibility exists that the further induction of PRP synthesis in the neuronal substrate shared by the three experiences may account for this improvement. Whichever the case is, our results demonstrate that after their reactivation, SOR and IA memories establish more tags than those capable of being satisfied by the protein synthesis induced during their reconsolidation. In fact, this is also implicit, and most likely the case, in the differential level of improvement induced by the exploration to a single novel OF but for different time periods.

It is worth considering that proteins captured by reconsolidation tags won’t be available for capture by the tags of the associated task (i.e., novelty). Thus, if proteins are limiting, the possibility exists of enhancing reconsolidation to the detriment of the associated task’s memory. Martinez and coworkers showed this phenomenon during memory consolidation^19^. Future research should evaluate this possibility in memory reconsolidation. But, its existence may have particular relevance in translational applications, especially if conserving the memory of the associated task holds interest.

In order to capture proteins, the reconsolidation tags should be available and functional^23,40–42^. Here, we reveal that exploring the novel arena immediately after a memory reactivation session affects a fundamental process that impairs the capture of the available proteins and therefore the reconsolidation. In terms of the behavioral tagging hypothesis, these results suggest the impairment of the reconsolidation-tags. The fact that the novel task may improve or impair a memory depending on the moment of its experiencing is both intriguing and expected. Experiments designed to study this process in memory consolidation demonstrated that the novel experience promotes LTM consolidation when it ensues around the hour of the training session, but not if it occurs too close to it (30 min before to immediately after)^11,18^. Considering the similarities between the behavioral tagging processes in memory consolidation, reconsolidation, and extinction, our results suggest that the exploration of a novel OF close to a weak training session did not promote IA and SOR memory consolidation because it may have impaired the learning-tags. A reciprocal interpretation of those results suggests the critical period for switching the novelty effects from improvement to impairment. The improving outcomes seem to occur until the 30 min before the tag setting and since the few minutes after it. The impairment properties shall emerge in between them. Yet, the differences observed between IA and SOR tasks suggest the need to identify specific time windows of improvement or impairment in a case-by-case evaluation.

As described previously, to reconsolidate, memories must first return to a labile state^9,43,44^. This phenomenon depends on specific processes triggered by the reactivation event^45–47^. Here, we showed that in the case of SOR and IA memories, the labilization depends on NMDA receptors. Moreover, disrupting memory labilization also impaired the improving effect of the associated novelty. This result highlights two relevant issues associated with the improvement of reconsolidated memories. First, the proteins synthesized by the novel OF reinforced the reconsolidation only if memories became labile. Second, it suggests that memory labilization is also associated with the setting of the reconsolidation-tags. Otherwise, if the tags had been set on non-labilazed memories, the novel experience should have enhanced their expression the next day.

This research provides a general mechanistic explanation of how and when behavioral events modulate memory by affecting the behavioral tagging process during its reconsolidation. However, identifying the specific machinery that motorizes the protein synthesis and the tag setting/maintenance processes is a pinnacle in the tagging and capture research at every level of study. Until now, works conducted in the IA showed that the tagging machinery at learning involves PKA (protein kinase A), CAMKII (Ca2 + /calmodulin-dependent protein kinase II), and BDNF/TrkB (Brain-derived neurotrophic factor/Tropomyosin receptor kinase B) signaling^23,40^. Unlike in the IA, ERK1/2 (Extracellular signal-regulated kinases 1/2) signaling contributes to setting the learning-tags in the SOR task^23,48^. These molecules, and other processes, also participate in the setting of synaptic tags required in long-term potentiation or depression, either at apical or basal dendrites in the pyramidal neurons of the hippocampus^40,42,49–51^. Despite the evidence indicating specific mechanisms associated with different tags in synaptic models of plasticity and behavioral tagging at learning, their whole nature remains foggy. Even foggier for the reconsolidation-tags due to the recency of their discovery. In this case, we showed that PKA supports the setting of SOR and IA reconsolidation-tags, while the ERK pathway is not involved in setting either of those^10^. Yet, we report here that the OF exploration enhanced memory reconsolidation during a wider time window in the SOR than in the IA task. This phenomenon suggests that their tag setting or tag maintenance machinery may enclose differences to unveil in future studies. Alternatively, a wider overlapping between novelty and SOR neuronal populations than between those of novelty and IA may explain these effects. It’s worth noting that the ERK pathway during SOR and IA reconsolidation is involved in the protein synthesis process^10^. This process also seems regulated by the noradrenergic system through the beta-adrenergic receptors of the hippocampus, at least in the reconsolidation of the event arena memory^21^. In fact, novelty activates adrenergic and dopaminergic systems, and they regulate protein synthesis in behavioral tagging during memory consolidation^23,25,52–55^. Thus, future studies should evaluate the possible role of the ventral tegmental area, the locus coeruleus, and their neurotransmission systems in regulating protein synthesis during the behavioral tagging process in memory reconsolidation.

Considering the potential benefits of modulating established memories for therapy and education, the reconsolidation process attracted considerable attention in human research. For instance, thorugh a translational research using the strategy implemented by Monfils and coworkers in rats^56^, Schiller and colleagues demonstrated that an extinction session shortly after fear memory reactivation could prevent its expression 24 h later^57^. Despite this fear-related memory was rendered in the lab, posterior research also evaluated this approach as a therapeutic strategy for addictions and phobias. Working with heroin addicts, Xue and colleagues showed that the exposure to a 45 min extinction session after the presentation of a heroin cue attenuated a cue-induced craving behavior that lasted at least six months^58^. Studies with hazardous drinkers and active smokers also showed similar effects when reactivation sessions were combined with extinction or counterconditioning therapies. In general, extinction and counterconditioning protocols were conducted closely afterward to the reactivation session. In the context of our work, this could be the result of alterations to the reconsolidation tags or competition for the available proteins^19^. Other studies evaluated the possibility of enhancing human memory during reconsolidation. Those assessed the sole effect of a reactivation session and also its eventual association with re-acquisition sessions, mild stressors, or physical exercise^33,35,36,59–61^. Our research along with Wang’s previous work^21^, emphasizes the significance of assessing the timing for associating behavioral events with reactivation sessions to enhance memory and persistence. Depending on the specific moment, these associated events could either disrupt the reconsolidation-tags, provide them with additional proteins, or compete for the available proteins. This dual effect, improvement or impairment, suggests that timing should be carefully studied when considering this strategy in education or clinical practice. Otherwise, combining reactivation cues with events at inappropriate time points could result in an enhanced instead of attenuated pathology. Equally, the opposite accounts for designing strategies to improve established memories.

In conclusion, our results reveal that activities performed within a critical time window around memory reactivation events can affect those memories by interacting with the fundamental mechanisms of the behavioral tagging process that underlay their reconsolidation. We think this should encourage using behavioral tagging-related protocols for designing non-invasive strategies to improve incremental learning in the educational field or to develop therapeutic strategies to attenuate maladaptive memories.

### Supplementary information

Supplementary Information

Reporting Summary

## Data Availability

Latencies and discrimination indexes for individual animals are available at https://osf.io/s5pj3/ in the Open Science Framework repository. All other data are available from the corresponding author on reasonable request.

## References

[1] McGaugh JL (2000). Memory–a century of consolidation. Science.

[2] Tronson NC, Taylor JR (2007). Molecular mechanisms of memory reconsolidation. Nat. Rev. Neurosci..

[3] Rossato, J. I. et al. State-dependent effect of dopamine D/D receptors inactivation on memory destabilization and reconsolidation. *Behav. Brain Res.*10.1016/j.bbr.2014.09.009 (2014). 10.1016/j.bbr.2014.09.009 25219363

[4] Morris RG (2006). Memory reconsolidation: sensitivity of spatial memory to inhibition of protein synthesis in dorsal hippocampus during encoding and retrieval. Neuron.

[5] Forcato C, Rodriguez ML, Pedreira ME, Maldonado H (2010). Reconsolidation in humans opens up declarative memory to the entrance of new information. Neurobiol. Learn Mem..

[6] Lee JL (2010). Memory reconsolidation mediates the updating of hippocampal memory content. Front. Behav. Neurosci..

[7] Sara SJ (2010). Reactivation, retrieval, replay and reconsolidation in and out of sleep: connecting the dots. Front. Behav. Neurosci..

[8] Misanin JR, Miller RR, Lewis DJ (1968). Retrograde amnesia produced by electroconvulsive shock after reactivation of a consolidated memory trace. Science.

[9] Nader K, Schafe GE, Le Doux JE (2000). Fear memories require protein synthesis in the amygdala for reconsolidation after retrieval. Nature.

[10] Rabinovich Orlandi I (2020). Behavioral tagging underlies memory reconsolidation. Proc. Natl. Acad. Sci. USA.

[11] Moncada D, Viola H (2007). Induction of long-term memory by exposure to novelty requires protein synthesis: evidence for a behavioral tagging. J. Neurosci..

[12] Morris RGM (2006). Elements of a neurobiological theory of hippocampal function: the role of synaptic plasticity, synaptic tagging and schemas. Eur. J. Neurosci..

[13] Frey U, Morris RG (1997). Synaptic tagging and long-term potentiation. Nature.

[14] Frey U, Morris RG (1998). Weak before strong: dissociating synaptic tagging and plasticity-factor accounts of late-LTP. Neuropharmacology.

[15] Moncada D, Ballarini F, Viola H (2015). Behavioral tagging: a translation of the synaptic tagging and capture hypothesis. Neural Plast..

[16] Moncada, D., Ballarini, F., Martinez, M. C. & Viola, H. In *Synaptic Tagging and Capture* (ed S. Sajikumar) Ch. 14, 231-259 (Springer New York, 2015).

[17] Redondo RL, Morris RGM (2011). Making memories last: the synaptic tagging and capture hypothesis. Nat. Rev. Neurosci..

[18] Ballarini F, Moncada D, Martinez MC, Alen N, Viola H (2009). Behavioral tagging is a general mechanism of long-term memory formation. Proc. Natl. Acad. Sci. USA.

[19] Martinez MC, Alen N, Ballarini F, Moncada D, Viola H (2012). Memory traces compete under regimes of limited Arc protein synthesis: implications for memory interference. Neurobiol. Learn Mem..

[20] Fonseca R, Nägerl UV, Morris RGM, Bonhoeffer T (2004). Competing for memory: hippocampal LTP under regimes of reduced protein synthesis. Neuron.

[21] Wang SH (2018). Novelty enhances memory persistence and remediates propranolol-induced deficit via reconsolidation. Neuropharmacology.

[22] Paxinos, G. & Watson, C. The rat brain in stereotaxic coordinates. *Acad. Pre*ss San Diego CA (1997).

[23] Moncada D, Ballarini F, Martinez MAC, Frey JU, Viola H (2011). Identification of transmitter systems and learning tag molecules involved in behavioral tagging during memory formation. Proc. Natl. Acad. Sci. USA.

[24] Vianna MR (2000). Role of hippocampal signaling pathways in long-term memory formation of a nonassociative learning task in the rat. Learn. Mem..

[25] Moncada D (2017). Evidence of VTA and LC control of protein synthesis required for the behavioral tagging process. Neurobiol. Learn Mem..

[26] Izquierdo I, hrîder N, Netto CA, Medina JH (1999). Novelty causes time-dependent retrograde amnesia for one-trial avoidance in rats through NMDA receptor- and CaMKII-dependent mechanisms in the hippocampus.. Eur. J. Neurosci..

[27] Winograd M, Viola H (2004). Detection of novelty, but not memory of spatial habituation, is associated with an increase in phosphorylated cAMP response element-binding protein levels in the hippocampus. Hippocampus.

[28] Dix SL, Aggleton JP (1999). Extending the spontaneous preference test of recognition: evidence of object-location and object-context recognition. Behav. Brain Res..

[29] Mumby DG, Gaskin S, Glenn MJ, Schramek TE, Lehmann H (2002). Hippocampal damage and exploratory preferences in rats: memory for objects, places, and contexts. Learn. Mem..

[30] Izquierdo I (2006). Different molecular cascades in different sites of the brain control memory consolidation. Trends Neurosci..

[31] Radiske A (2017). Prior learning of relevant nonaversive information is a boundary condition for avoidance memory reconsolidation in the rat hippocampus. J. Neurosci..

[32] Correa J, Tintorelli R, Budriesi P, Viola H (2022). Persistence of spatial memory induced by spaced training involves a behavioral-tagging process. Neuroscience.

[33] Forcato C, Rodriguez ML, Pedreira ME (2011). Repeated labilization-reconsolidation processes strengthen declarative memory in humans. PLoS One.

[34] Lee JL (2008). Memory reconsolidation mediates the strengthening of memories by additional learning. Nat. Neurosci..

[35] Fukushima H (2014). Enhancement of fear memory by retrieval through reconsolidation. Elife.

[36] Tassone LM (2020). Memory reconsolidation as a tool to endure encoding deficits in elderly. PLoS One.

[37] Jones BJ, Chen ME, Simoncini L, Spencer RMC (2022). Sleep enhances reconsolidation-based strengthening of visuospatial memories. Sci. Rep..

[38] Nomoto M (2016). Cellular tagging as a neural network mechanism for behavioural tagging. Nat. Commun..

[39] Guzowski JF, McNaughton BL, Barnes CA, Worley PF (1999). Environment-specific expression of the immediate-early gene Arc in hippocampal neuronal ensembles. Nat. Neurosci..

[40] Lu Y (2011). TrkB as a potential synaptic and behavioral tag. J. Neurosci..

[41] Sajikumar S, Frey JU (2004). Resetting of ‘synaptic tags’ is time- and activity-dependent in rat hippocampal CA1 in vitro. Neuroscience.

[42] Sajikumar S, Navakkode S, Frey JU (2007). Identification of compartment- and process-specific molecules required for “synaptic tagging” during long-term potentiation and long-term depression in hippocampal CA1. J. Neurosci..

[43] Merlo E, Milton AL, Goozee ZY, Theobald DE, Everitt BJ (2014). Reconsolidation and extinction are dissociable and mutually exclusive processes: behavioral and molecular evidence. J. Neurosci..

[44] Pedreira ME, Perez-Cuesta LM, Maldonado H (2004). Mismatch between what is expected and what actually occurs triggers memory reconsolidation or extinction. Learn. Mem..

[45] Ben Mamou C, Gamache K, Nader K (2006). NMDA receptors are critical for unleashing consolidated auditory fear memories. Nat. Neurosci..

[46] Lee SH (2008). Synaptic protein degradation underlies destabilization of retrieved fear memory. Science.

[47] Suzuki A, Mukawa T, Tsukagoshi A, Frankland PW, Kida S (2008). Activation of LVGCCs and CB1 receptors required for destabilization of reactivated contextual fear memories. Learn. Mem..

[48] Tintorelli R (2020). Spatial-memory formation after spaced learning involves ERKs1/2 activation through a behavioral-tagging process. Sci. Rep..

[49] Redondo RL (2010). Synaptic tagging and capture: differential role of distinct calcium/calmodulin kinases in protein synthesis-dependent long-term potentiation. J. Neurosci..

[50] Ishikawa Y, Horii Y, Tamura H, Shiosaka S (2008). Neuropsin (KLK8)-dependent and -independent synaptic tagging in the Schaffer-collateral pathway of mouse hippocampus. J. Neurosci..

[51] Fonseca R (2012). Activity-dependent actin dynamics are required for the maintenance of long-term plasticity and for synaptic capture. Eur. J. Neurosci..

[52] Takeuchi T (2016). Locus coeruleus and dopaminergic consolidation of everyday memory. Nature.

[53] Lisman JE, Grace AA (2005). The hippocampal-VTA loop: controlling the entry of information into long-term memory. Neuron.

[54] Kitchigina, V., Vankov, A., Harley, C. & Sara, S. J. Novelty-elicited, noradrenaline-dependent enhancement of excitability in the dentate gyrus *Eur. J. Neurosci.* **9**, 41–47 (1997). 10.1111/j.1460-9568.1997.tb01351.x 9042567

[55] Duszkiewicz AJ, McNamara CG, Takeuchi T, Genzel L (2019). Novelty and dopaminergic modulation of memory persistence: a tale of two systems. Trends Neurosci..

[56] Monfils MH, Cowansage KK, Klann E, LeDoux JE (2009). Extinction-reconsolidation boundaries: key to persistent attenuation of fear memories. Science.

[57] Schiller D (2010). Preventing the return of fear in humans using reconsolidation update mechanisms. Nature.

[58] Xue YX (2012). A memory retrieval-extinction procedure to prevent drug craving and relapse. Science.

[59] Coccoz V, Maldonado H, Delorenzi A (2011). The enhancement of reconsolidation with a naturalistic mild stressor improves the expression of a declarative memory in humans. Neuroscience.

[60] Tay KR, Flavell CR, Cassini L, Wimber M, Lee JLC (2019). Postretrieval relearning strengthens hippocampal memories via destabilization and reconsolidation. J. Neurosci..

[61] Keyan D, Bryant RA (2017). Acute physical exercise in humans enhances reconsolidation of emotional memories. Psychoneuroendocrinology.

