# Supplementary material for: Modulation of memory reconsolidation by adjacent novel tasks: timing defines the nature of change
Source: Commun Biol. 2023 Dec 19;6:1288. doi: 10.1038/s42003-023-05666-5 (PMC10730840; doi:10.1038/s42003-023-05666-5)
Supplement: Supplementary file 1 — Supplementary Information [file 42003_2023_5666_MOESM1_ESM.pdf]

Supplementary Information. Modulation of memory reconsolidation by adjacent novel tasks: timing defines the nature of change

Supplementary Methods.

Familiar OF: We familiarized the animals with the OF by exposing them to the arena for 30 min for 2 consecutive days. We and others showed that this exposure time induces spatial familiarization [1-4].

Novel OF exploration times: We submitted animals to explore a novel OF for either 2 or 5 min. Both times are sufficient to detect spatial novelty [5,6].

Supplementary Note 1.

Results corresponding to supplementary figure 1:

Previous evidence obtained from memory consolidation experiments shows that exploring an OF promoted LTM formation through a behavioral tagging mechanism if the arena was novel, but not if it was familiar. Moreover, this novelty-related effect was associated with a higher overlap between the neuronal population activated by the learned task (Novel object recognition) and the exploration of the arena[7]. Therefore, we decided to evaluate the requirement of the novel nature of the arena to improve SOR and IA LTM during their reconsolidation. To do so, we familiarized a group of animals with the OF. The familiarization sessions were separated for at least 3 h from the habituation to the contexts used for the SOR or IA tasks. On day 3 we trained animals in the correspondent task (SOR or IA), without exposing them to the OF. On day 4, 60 min before submitting them to a memory reactivation session, we exposed the animals to an OF for 5 min. Half of them had been familiarized with the arena. We submitted a third group of animals to a memory reactivating session but without exposing them to any OF. On day 5 we evaluated the memory expression of the three groups on the corresponding memory task. As shown in supplementary figure 1 the exploration of a novel OF 60 min before the reactivation session improved LTM expression at the next day. On the contrary, the animals that explored a familiar showed levels of memory equivalent to those of animals that did not explore any OF. These results were equivalent in both SOR (Supplementary Figure 1a) and IA (Supplementary Figure 1b) tasks.

Supplementary Note 2.

Results corresponding to supplementary figure 2:

We evaluated a possible effect of the time of exploration to a novel OF on the improvement of SOR and IA memory during their reconsolidation. To do so, we trained animals in either the SOR or the IA tasks, as described in the main text. On day 4, the animals explored a novel OF for 2 or 5 min 60 before submitting them to a memory reactivation session in the corresponding task. A third group of animals (control) experienced the reactivation session but did not explore any OF. 24 h later we evaluated the memory and observed higher levels of expression in those animals that explored a novel OF. Surprisingly the animals that explored the OF for 5 min outperformed those who did it for only 2 min. This effect was equivalent in both SOR (Supplementary Figure 2a) and IA (Supplementary Figure 2b) tasks.

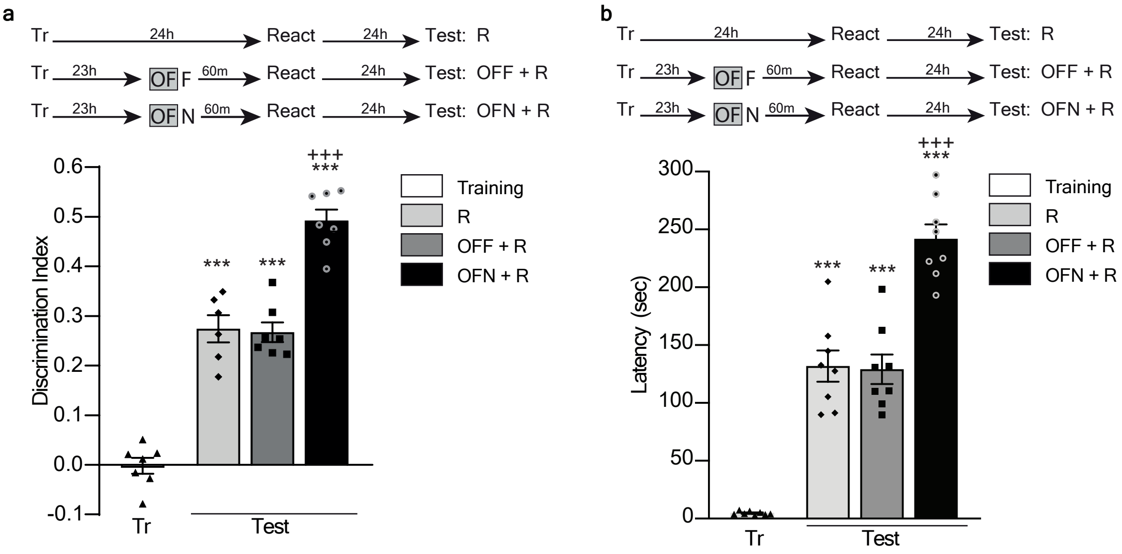

Supplementary Figure 1: Pre-reactivation exposure to a novel but not a familiar OF improves LTM during reconsolidation.

Top: experimental design. Tr: training. R: animals exposed to a memory reactivation session. OFF: animals that explored a familiar OF 60 min before a memory reactivation session. OFN: animals that explored a novel OF 60 min before a memory reactivation session.

a) Figure shows the discrimination index between the object moved to the novel position and the non-moved object, expressed as mean±SEM, during training and test sessions in the SOR task. *** $p<0.001$ vs Tr. +++ $p<0.001$ vs R and OFF + R. Multiple comparison after one-way ANOVA (n=6-7). **b)** Figure shows the latency to step-down from the platform, expressed as mean±SEM, during training and test sessions in the IA task. *** $p<0.001$ vs Tr. +++ $p<0.001$ vs R and OFF + R. Multiple comparison after Welch's ANOVA (n=8).

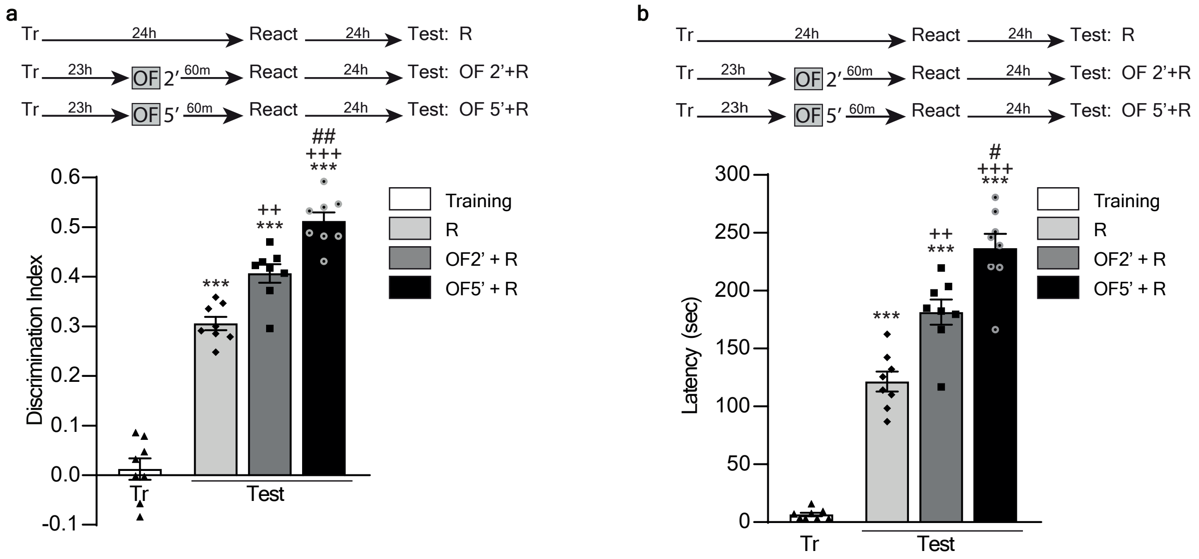

Supplementary Figure 2. Differential improvement of LTM during reconsolidation as a result of the exploration time to a novel OF.

Top: experimental design. Tr: training. R: animals exposed to a memory reactivation session. OF2' + R: animals that explored a novel OF during 2 min, 60 min before memory reactivation session. OF5' + R animals that explored the novel OF during 5 min, 60 min before the memory reactivation session

a) Figure shows the discrimination index between the object moved to the novel position and the non-moved object, expressed as mean±SEM, during training and test sessions in the SOR task. *** $p<0.001$ vs Tr. +++, ++ $p<0.001$ and $p<0.01$ vs R. ## $p<0.01$ vs OF2' + R group. Multiple comparison after one-way ANOVA (n=8). **b)** Figure shows the latency to step-down from the platform, expressed as mean±SEM, during training and test sessions in the IA task. *** $p<0.001$ vs Tr. +++, ++ $p<0.001$ and $p<0.01$ vs R. # $p<0.05$ vs OF2' + R. Multiple comparison after Welch's ANOVA (n=8)

Supplementary References

1 Moncada, D. & Viola, H. Phosphorylation state of CREB in the rat hippocampus: a molecular switch between spatial novelty and spatial familiarity? *Neurobiol Learn Mem* **86**, 9-18 (2006).

2 Moncada, D. & Viola, H. PKMzeta inactivation induces spatial familiarity. *Learning & memory (Cold Spring Harbor, N.Y.)* **15**, 810-814 (2008).

3 de Carvalho Myskiw, J., Benetti, F. & Izquierdo, I. Behavioral tagging of extinction learning. *Proc Natl Acad Sci USA* **110**, 1071-1076, doi:10.1073/pnas.1220875110 (2013).

4 Vishnoi, S., Raisuddin, S. & Parvez, S. Behavioral Tagging: Role of Neurotransmitter Receptor Systems in Novel Object Recognition Long-Term Memory. *ACS Omega* **7**, 11587-11595, doi:10.1021/acsomega.1c05865 (2022).

5 Winograd, M. & Viola, H. e. Detection of novelty, but not memory of spatial habituation, is associated with an increase in phosphorylated cAMP response element-binding protein levels in the hippocampus. *Hippocampus* **14**, 117-123 (2004).

6 Izquierdo, I., hrîder, N., Netto, C. A. & Medina, J. H. Novelty causes time-dependent retrograde amnesia for one-trial avoidance in rats through NMDA receptor- and CaMKII-dependent mechanisms in the hippocampus. *The European journal of neuroscience* **11**, 3323-3328 (1999).

7 Nomoto, M. *et al.* Cellular tagging as a neural network mechanism for behavioural tagging. *Nat Commun* **7**, 12319, doi:10.1038/ncomms12319 (2016).
